# Risk factors of cervical lymph node metastasis in multifocal papillary thyroid cancer

**DOI:** 10.3389/fonc.2022.1003336

**Published:** 2022-12-08

**Authors:** Ting Zhang, Liang He, Zhihong Wang, Wenwu Dong, Wei Sun, Ping Zhang, Hao Zhang

**Affiliations:** Department of Thyroid Surgery, The First Hospital of China Medical University, Shenyang, Liaoning, China

**Keywords:** multifocal PTC, risk factors, central lymph node metastasis, lateral lymph node metastasis, axial diameters

## Abstract

**Introduction:**

Identifying risk variables for cervical lymph node metastases in multifocality papillary thyroid cancer (MPTC) could assist surgeons in determining whether cervical lymph node dissection would be an appropriate surgical option.

**Methods:**

A retrospective cohort of 2006 patients with papillary thyroid cancer were selected. MPTC (N = 460) was defined as the presence of two or more foci of PTC. The risk factors for central lymph node metastasis (CLNM) and lateral lymph node metastasis (LLNM) in MPTC were investigated by univariate and multivariate analyses, including the following items: age at diagnosis, gender, Hashimoto’s thyroiditis, extrathyroidal extension (ETE), maximal axial diameter (MAD) and the sum of axial diameters (SAD) of tumor. In addition, CLNM was used to evaluate LLNM.

**Results:**

The incidence of CLNM and LLNM was 44.57% and 17.17%, respectively. The multivariate analysis demonstrated that gender, extrathyroidal extension (ETE), age, maximal axial diameter (MAD), and the sum of axial diameters (SAD) were related to increased risk for CLNM in MPTC (*p* < 0.05). The area under the receiver operating characteristic (ROC) curve (AUC) for age at diagnosis of CLNM was 0.647, the cut-off value was 50 years old. Additionally, by multivariate analysis, CLNM, ETE, MAD, and SAD were independent risk factors for LLNM in MPTC (*p* < 0.05). ROC curve analysis demonstrates that AUC for MAD and SAD diagnosis of LLNM were 0.639 and 0.757, and the cut-off values were 16 and 26 mm, respectively.

**Conclusions:**

MPTC patients who have risk factors for CLNM were advised to perform prophylactic central lymph node dissection (CLND). Additionally, the presence of risk factors for LLNM should be individually evaluated and analyzed for the necessity of lateral lymph node dissection.

## Introduction

Papillary thyroid cancer (PTC) is the most common thyroid malignancy with a high tendency of lymph node metastasis (1). PTC is characterized by multifocality. Multifocal PTC (MPTC) is defined as PTC with two or more anatomically separate (noncontiguous) foci within the thyroid gland that involve either a single thyroid lobe (unilateral disease) or both lobes (bilateral disease) (2). Clinically, the prevalence of MPTC ranges from 18% to 87%, depending on epidemiological factors (3). The condition has a decent prognosis, with surgery as the primary treatment option.

Nevertheless, it is well-established that PTC has a strong tendency for lymph node metastasis. Approximately 20% to 90% of PTC patients will have clinical or occult cervical lymph node involvement (4). Lymph node metastasis in PTC follows a predictable pattern. Most commonly, tumor cells typically metastasize to lymph nodes of the central neck compartment (level VI), followed by those of the lateral neck (levels II, III, and IV) (5). Current knowledge suggests that cervical lymph node metastasis is the most crucial variable to increase the risk of local recurrence and overall survival (6). Our recent study (7) and studies by others (8, 9) have suggested that MPTC increases the risk of cervical lymph node metastasis. Cervical lymph node metastasis is often accompanied with a poor prognosis, including distant metastasis and persistent or recurrent disease.

Due to the increased incidence of complications such as recurrent laryngeal nerve injury, hypoparathyroidism, and others in the first operation or reoperation of central lymph node dissection (CLND) in recurring cases, it remains controversial whether CLND should be performed routinely in the initial operation (10, 11). The American Thyroid Association (ATA) recommends therapeutic cervical lymph node dissections in patients with clinical evidence of lymph node involvement. Although prophylactic cervical lymph node dissection is recommended, its usefulness remains largely controversial because of the lack of high-quality, evidence-based study (4).

Identifying the risk factors of cervical lymph node metastasis in MPTC could help surgeons evaluate the clinical status of individuals with PTC and determine whether CLND or/and lateral lymph node dissection (LLND) would be an appropriate surgical option (12, 13). However, few studies have evaluated lymph node metastasis in MPTC, especially the risk factors of cervical lymph node metastasis in MPTC are rarely mentioned. This study performed a retrospective analysis to identify the risk factors of central lymph node metastasis (CLNM) and lateral lymph node metastasis (LLNM) in MPTC. The results of the study will help inform clinical decision-making.

## Materials and methods

### Data collection

This retrospective analysis was conducted in PTC patients (N = 2006) who underwent initial surgery from January 1, 2016, to December 31, 2016, at the Department of Thyroid Surgery, the First Hospital of China Medical University. The study included patients who met the following criteria at initial thyroid surgery: all patients had a preoperative examination. Those who underwent neck surgery, radiation exposure, a definite or suspected family history of PTC, and incomplete medical records were excluded. Patients who underwent thyroidectomy or total thyroidectomy with ipsilateral or bilateral central compartment. Additionally, lateral neck dissection was performed during surgery for N1b patients.

In the present study, MPTC (N = 460) was defined as the presence of two or more foci of PTC. For patients with multifocality, each focus was recorded separately. All patients had a preoperative examination (within 1 week), including thyroid and neck lymph node ultrasound, enhanced neck computed tomography, preoperative fine-needle aspiration biopsy (some patients), and Tg detection of lymph node aspiration biopsy eluent (some patients). We can preliminarily evaluate whether it is a single focus or multiple focus, and lymph node is metastasis or not. In addtion, intraoperative frozen section (tumor focus and central lymph node) were confirmed the diagnosis of the multifocal tumor and/or CLNM during primary operation. Therefore, we perform prophylactic central lymph node dissection (CLND) in all PTC patients and selective latal lymph node dissection (LLND) in LLNM. The histologic diagnosis was classified according to the World Health Organization system. Specimen were reviewed by two experienced pathologists in a blinded fashion, who confirmed the diagnosis of PTC and the number of tumor foci. Two senior pathologists used a systematic technique to determine the presence or absence of lymph node metastases in postoperative pathology findings. Postoperative pathology was also used to identify Hashimoto’s thyroiditis.

We analyzed the risk factors for CLNM, including the following items: age at diagnosis, gender, Hashimoto’s thyroiditis, ETE, tumor diameter, including maximal axial diameter (MAD) and the sum of axial diameters (SAD) of tumor, respectively. SAD refers to the sum of all tumor focus axial diameters in MPTC. In addition to the above clinical features and tumor histological characteristics, CLNM and LLNM was evaluated.

### Statistical analysis

The data were analyzed through IBM SPSS 23.0 statistical software package (SPSS Inc., Chicago, IL, USA). Categorical variables were presented as the number of observations and percentages, while continuous variables were expressed as means ± standard deviation. Univariate analysis was performed using chi-square tests or Fisher exact tests for categorical variables. Student’s t-test was used for normally distributed data, and Mann–Whitney U-test was used for continuous variables that were not normally distributed. Multivariate logistic regression analyses were utilized to confirm the results obtained for independent predictive factors associated with the risk of CLNM and LLNM in MPTC. The diagnostic potential of independent variables was determined by receiver operating characteristic (ROC) analyses and was expressed as the area under ROC curve (AUC). The sensitivity, specificity, and Youden index were also calculated. All values with a probability (*p*) < 0.05 were considered statistically significant.

## Results

A total of 2006 patients with PTC were selected for the study. Of these, 460 have multifocal PTC and were included in the study. Multifocal PTC is more likely to have CLNM and LLNM than unifocal PTC. The incidence of CLNM and LLNM was 33.05% (511/1546) and 9.89% (153/460) in unifocal PTC. However, the incidence is 44.57% (205/460) and 17.17% (79/460) in MPTC. There were 79 patients with LLNM, and 10 (2.17%) had no CLNM.

**Figure 1:**
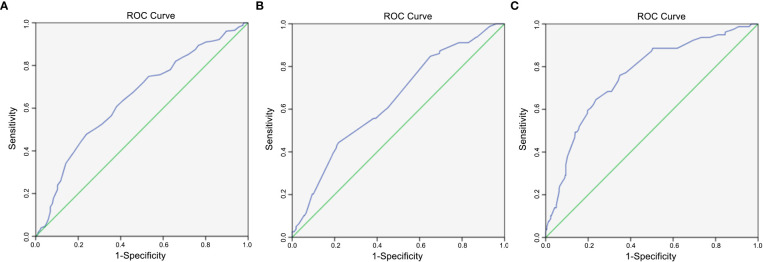
**(A)** Diagnostic value of age. The area under ROC curve was 0.647 (95% confidence interval = 0.597–0.697, *p* < 0.005); **(B)** Diagnostic value of maximal axial diameter (MAD). The area under ROC curve was 0.639 (95% confidence interval = 0.573–0.705, *p* < 0.005); **(C)** Diagnostic value of sum of axial diameters (SAD). The area under ROC curve was 0.757 (95% confidence interval = 0.699–0.815, *p* < 0.005).

We divided the 460 patients into two groups based on CLNM: 255 without CLNM and 205 with CLNM (**Table 1**). On the univariate analysis, gender, ETE, mean age, MAD and SAD were significantly correlated with CLNM (*p* < 0.05; **Table 2**). However, Hashimoto’s thyroiditis is not significant. To determine how strongly those factors were associated with CLNM, the multivariable logistic regression model was performed. It was demonstrated that gender (OR, 0.482; 95% CI, 0.293–0.792), ETE (OR, 0.325; 95% CI, 0.182–0.579), and age (OR, 1.049; 95% CI, 1.029–1.069) but MAD (OR, 0.988; 95% CI, 0.939–1.039), and SAD (OR, 0.982; 95% CI, 0.95–1.014) were related to increased risk for MPTC (p < 0.05; **Table 3**). Because age is a continuous variable, ROC curve analysis revealed that the AUC for age at diagnosis of CLNM was 0.647. The cut-off value was 50 years old, and the sensitivity and specificity were 47.8% and 76.1%, respectively, with a Youden index of 0.239 (**Figure 1A**).

**Table 1:** Number of CLNM and LLNM in MPTC.

		CLNM		*p*-value	LLNM		*p*-value
		+	-		+	-	
Multifocality	+	205	255	0.000	79	381	0.000
	–	511	1035		153	1393	

central lymph node metastasis (CLNM), lateral lymph node metastasis (LLNM).

**Table 2:** Univariate analysis of the clinical and pathological factors associated with CLNM.

	CLNM		*p*-value
	+	-	
Gender			0.000
Male	56	36	
Female	149	219	
Hashimoto’s thyroiditis			0.145
Yes	81	118	
No	124	137	
ETE			0.000
+	45	23	
–	160	232	
Age	42.11 ± 11.18	47.50 ± 10.55	0.000
MAD	15.93 ± 9.49	12.50 ± 8.35	0.000
SAD	26.00 ± 14.63	20.59 ± 13.05	0.000

central lymph node metastasis (CLNM), extrathyroidal extension (ETE), maximal axial diameter (MAD), the sum of axial diameters (SAD).

**Table 3:** Multivariate analysis of factors associated with CLNM.

	SE	χ^2^	*p*-value	OR	95% CI	
Gender (male)	0.254	8.286	0.004	0.482	0.293	0.792
ETE	0.295	14.538	0	0.325	0.182	0.579
Age	0.01	23.776	0	1.049	1.029	1.069
MAD	0.026	0.238	0.626	0.988	0.939	1.039
SAD	0.017	1.255	0.263	0.982	0.95	1.014
constant	0.474	4.492	0.034	0.366	–	–

central lymph node metastasis (CLNM), extrathyroidal extension (ETE), maximal axial diameter (MAD), the sum of axial diameters (SAD).

The 460 patients were also divided into two groups based on LLNM: 381 without LLNM and 79 with LLNM (**Table 1**). On univariate analysis, gender, ETE, age, CLNM, MAD and SAD were significantly correlated with LLNM (*p* < 0.05; **Table 4**). However, Hashimoto’s thyroiditis wss insignificant. To determine how strongly those factors were associated with LLNM, the multivariable logistic regression model was performed. The result showed that CLNM (OR, 0.118; 95% CI, 0.055–0.251), ETE (OR, 0.388; 95% CI, 0.193–0.778), MAD (OR, 1.125; 95% CI, 1.06–1.194), and SAD (OR, 0.89; 95% CI, 0.855–0.926) but gender (OR, 0.665; 95% CI, 0.338–1.308), and age (OR, 1.017; 95% CI, 0.99–1.044) were an independent risk factor for LLNM in MPTC (*p* < 0.05; **Table 5**). Because MAD and SAD were continuous variable, ROC curve analysis indicated that the AUC for MAD (**Figure 1B**) and SAD (**Figure 1C**) at diagnosis of LLNM were 0.639 and 0.757, respectively. The cut-off values were 16 and 26 mm. The sensitivity and specificity for MAD diagnosis of LLNM were 44.3% and 78.2%, with a Youden index of 0.225. The sensitivity and specificity for SAD diagnosis of LLNM were 64.6% and 76.4%, with a Youden index of 0.41, respectively.

**Table 4:** Univariate analysis of the clinical and pathological factors that could be associated with LLNM.

	LLNM		*p*-value
	+	-	
Gender			0.011
Male	24	68	
Female	55	313	
Hashimoto’s thyroiditis			0.529
Yes	36	159	
No	43	222	
ETE			0.001
+	25	43	
–	54	338	
CLNM			0.000
+	69	136	
–	10	245	
age	43.73 ± 11.57	46.20 ± 10.69	0.018
MAD	17.27 ± 11.72	12.88 ± 7.89	0.002
SAD	33.45 ± 16.34	21.00 ± 11.86	0.000

lateral lymph node metastasis (LLNM), central lymph node metastasis (CLNM), extrathyroidal extension (ETE), maximal axial diameter (MAD), the sum of axial diameters (SAD).

**Table 5:** Multivariate analysis of factors associated with LLNM.

	SE	χ^2^	*p*-value	OR	95% CI	
Gender (male)	0.345	1.398	0.237	0.665	0.338	1.308
ETE	0.355	7.106	0.008	0.388	0.193	0.778
CLNM	0.387	30.587	0	0.118	0.055	0.251
Age	0.013	1.548	0.213	1.017	0.99	1.044
MAD	0.03	15.06	0	1.125	1.06	1.194
SAD	0.02	33.506	0	0.89	0.855	0.926
constant	0.776	24.686	0	47.165	–	–

lateral lymph node metastasis (LLNM), extrathyroidal extension (ETE), maximal axial diameter (MAD), the sum of axial diameters (SAD).

## Discussion

Although PTC is generally thought to be a tumor with an excellent prognosis, some studies described patients with very aggressive or fatal cases of MPTC (14, 15). CLNM and LLNM are common in MPTC, and surgically overlooked cervical lymph node metastasis is a major cause of tumor recurrence (16). It is well-established that PTC has a high tendency for cervical lymph node metastasis. Up to one-third of patients have clinically detectable lymph node involvement on initial presentation (17). Of patients with no detectable nodal disease on examination, an estimated 80% will have micro metastatic lymph node disease on postoperative pathologic examination (18). Lymph node metastasis in PTC increases the chance of local disease recurrence (19).

Although many studies have identified nodal disease as a poor prognostic factor for long-term patient survival, this remains controversial (20, 21). Some scholars (22–25) advocate routine CLND to prevent a future recurrence. It was reported that removing positive lymph nodes and accurate staging are critical for the initial surgery, which is closely associated with reducing postoperative thyroglobulin (Tg) levels, and a lower morbidity rate. However, other studies (26, 27) suggest that this procedure increases the rate of complications, without providing any demonstrable benefits in terms of long-term survival. Our previous studies confirmed that multifocality of PTC is a risk factor of disease recurrence and poor prognosis. There is a higher incidence of lymph node metastasis in multifocal PTC than unifocal PTC. Predictive factors for CLNM and LLNM in MPTC might be helpful for clinicians to evaluate the clinicopathological status of PTC and help surgeons for clinical decision-making (7).

This study focuses on the association of MPTC with CLNM and LLNM. It was revealed that CLNM and LLNM rate were 44.57% and 17.17% in MPTC, respectively. Several studies have reported risk factors of CLNM associated with multifocality and bilaterality, but few studies evaluated these characteristics concerning the recommended surgical procedure. In recent studies, the prevalence of CLNM was 8% - 77% in MPTC (8, 28, 29), and 17% - 61% in LLNM (30, 31), which were similar to our results. Thus, CLND should be considered, and LLND must be evaluated carefully, particularly in patients with high-risk factors or suspected metastasis during the preoperative assessment in MPTC.

The presence of CLNM is significantly associated with gender, ETE, age, and tumor diameter in MPTC. Most studies (32–34) indicated that males, ETE, and young people are independent risk factors of CLNM. In our study, the proportion of CLNM in females was substantially lower than that in males. In addition, multivariate analysis confirmed that male is an independent risk factor of CLNM. Similarly, univariate and multivariate analyses demonstrated that ETE and young age were also significantly associated with CLNM in MPTC. The prevalence of CLNM was 60.87% (56/92) in males and 40.49% (149/368) in females. The prevalence of CLNM was 66.18% (45/68) in ETE and 40.82% (160/392) in non-ETE. The average age of CLNM and non-CLNM groups are 42.11 and 47.50, respectively. According to our study, for patients with age ≤ 50, the specificity and sensitivity were 76.1% and 47.8%, respectively. As the age decreased, the prevalence of CLNM rapidly increased, which provides reference for the decision on whether to perform CLND. On the other hand, recurrent laryngeal nerve injury and hypoparathyroidism may happen during operation, and CLND should be avoided when the risk factors of CLNM are excluded in MPTC.

We distinguished the clinical risk factors for LLNM in MPTC. Some authors suggested that gender and age are significant risk factors for LLNM in MPTC (35, 36). In our study, univariate analysis showed that gender, age, CLNM, ETE, MAD and SAD were classified as risk factors of LLNM in MPTC. Unfortunately, CLNM, ETE, MAD and SAD, not gender and age, were independent risk factors for the presence of LLNM by multivariate analysis. According to our study, when MAD ≥ 16 mm, the specificity and sensitivity were 78.2% and 44.3%, respectively. Similarly, when SAD ≥ 26 mm, the specificity and sensitivity were 76.4% and 64.6%, respectively. Moreover, the prevalence of LLNM rapidly increased with increasing MAD or SAD. Consequently, clinicians should choose more aggressive initial treatment and closer follow-up for MPTC with MAD ≥ 16 mm or SAD ≥ 26 mm, and more attention should be paid to LLNM and recurrence/persistence during the follow-up.

The present study discovered that ETE is a risk factor for CLNM and LLNM in MPTC. However, Hashimoto’s thyroiditis was not classified as a risk factor for CLNM and LLNM, which was inconsistent with published results (37, 38). This discrepancy might be caused by differences in the selection criteria and study designs. The univariate analysis revealed that MAD and SAD differed between CLNM and non-CLNM in MPTC. Still, we did not observe any difference between CLNM and non-CLNM in multivariate analysis.

Similarly, the univariate analysis showed that gender and age differed between LLNM and non-LLNM in MPTC. Nonetheless, we did not observe any difference between LLNM and non-LLNM in multivariate analysis, possibly due to the small sample size in one year, which will be increased in future studies.

To the best of our knowledge, this is the first study that reported that age at diagnosis is associated with increased CLNM susceptibility and tumor diameter increased LLNM susceptibility in MPTC. Our results revealed that the mean age of CLNM was lower than that of non CLNM in MPTC. Additionally, MAD and SAD of LLNM were more significant than that of non LLNM in MPTC. ROC curve revealed that age and tumor diameter have potential diagnostic significance in CLNM and LLNM in MPTC. Thus, combined use of clinicopathological biomarkers with other imaging techniques and fine-needle aspiration cytological results should be used to optimize the diagnosis in MPTC.

There are several limitations in this study. 1) We did not use other diagnostic techniques such as FNA to evaluate lymph node characteristics. We will analyze such specimens as FNA is used routinely in our clinical practice. 2) We did not perform elective lateral lymph node dissection in all PTMC patients. 3) We did not investigate the association between the number of foci detected and CLNM and LLNM in MPTC. Only patients with highly suspicious imaging evidence or cytologically confirmed lateral lymph node metastasis had lateral lymph node dissection performed. Finally, our study was constrained by its retrospective design and limited number of MPTC. Another limitation of our study was its single-center analysis. Multicenter and large cohort studies should be performed to validate the risk factors for MPTC.

## Conclusions

Our study identified the risk factors of CLNM and LLNM in MPTC patients. MPTC patients who are male and of young age at preoperative evaluation, or ETE during the intraoperative evaluation were recommended to perform prophylactic CLND, particularly in those patients with age ≤50. The presence of ETE, CLNM, a larger size MAD (≥ 16 mm), or SAD (≥ 26 mm) could help surgeons evaluate the lateral lymph node status of MPTC patients and further analyze the necessity of LLND individually for these patients.

## Data availability statement

The raw data supporting the conclusions of this article will be made available by the authors, without undue reservation.

## Ethics statement

The study protocol was approved by the Institutional Review Board of the First Affiliated Hospital of China Medical University and was in compliance with the Helsinki Declaration (AF-SOP-07-1.0-01). The same group of surgeons performed all procedures. The patients/participants provided their written informed consent to participate in this study. Written informed consent was obtained from the individual(s) for the publication of any potentially identifiable images or data included in this article.

## Author contributions

HZ: substantial contributions to the conception or design of the work. TZ: drafting the work or revising it critically for important intellectual content. LH: the acquisition of data for the work. WS: the analysis of data for the work. ZW: the acquisition of data for the work. WD: the interpretation of data for the work. PZ: final approval of the version to be published. All authors contributed to the article and approved the submitted version.
